# Differential Effects of Dietary Oils on Emotional and Cognitive Behaviors

**DOI:** 10.1371/journal.pone.0120753

**Published:** 2015-03-23

**Authors:** Keiko Kato

**Affiliations:** Faculty of Life Sciences, Kyoto Sangyo University, Kyoto, Japan; NIMH, NIH, UNITED STATES

## Abstract

Several dietary oils have been used preventatively and therapeutically in the setting of neurological disease. However, the mechanisms underlying their influence on brain function and metabolism remain unknown. It was investigated whether 3 types of dietary oils affected emotional behaviors in mice. Wild-type (WT) mice and sialyltransferase ST3Gal IV-knockout (KO) mice, which exhibit increased emotional and cognitive behaviors, were fed diets containing 20% dietary oils from post-weaning to adulthood. Mice were fed pellets made from control feed AIN93G powder containing 18% fish oil, soybean oil, or a mixture of 1-palmitoyl-2-oleoyl-3-palmitoyl glycerol (POP) and 1-stearoyl-2-oleoyl-3-stearoyl glycerol (SOS), plus 2% soybean oil. Once mice reached adulthood, they were subjected to fear conditioning test to measure cognitive anxiety and forced swim test to measure depression. WT mice fed the POP-SOS diet showed a 0.6-fold decrease in percent freezing with contextual fear compared with WT mice fed the control diet. KO mice fed the fish oil diet showed a 1.4-fold increase in percent freezing with contextual fear compared with KO mice fed the control diet. These findings indicate that response to contextual fear was improved in WT mice that consumed POP-SOS but aggravated in KO mice that consumed fish oils. Furthermore, KO mice showed a 0.4-fold decrease in percent freezing in response to tone fear when they were fed POP-SOS diet compared to a control diet. Thus, POP-SOS diet reduced tone fear level of KO mice until the same level of WT mice. Finally, KO mice fed the soybean oil diet showed a 1.7-fold increase in immobility in the forced swim test compared to KO mice fed the control diet. Taken together, oil-rich diets differentially modulate anxiety and depression in normal and anxious mice. Oils rich in saturated fatty acids may alleviate anxiety more strongly than other oils.

## Introduction

It has long been known that dietary fatty acids improve some limbic and cortical functions in humans. Ketogenic diets use long-chain fatty acids (LCFAs, ≥ 16 carbon atoms) including palmitic acid and stearic acid and/or medium-chain triglycerides (MCTs, 8–14 carbon atoms) to improve seizures in patients with intractable epilepsy [1,2]. Clinical trials have shown that n-3 polyunsaturated fatty acids, especially eicosapentaenoic acid (EPA), can reduce depression [3]. Similarly, experimental trials have demonstrated that an n-3 polyunsaturated fatty acid deficiency, especially docosahexaenoic acid (DHA) deficiency, can increase depression- and anxiety-like behaviors [4–6]. However, an epidemiological report has shown that vegetarian diets including only triglyceride oils composed of alpha-linolenic (n-3 fatty acid) and linoleic acids (n-6 fatty acid) maintain healthy mood states [7] more effectively than diets including fish oils composed of DHA and EPA [8]. These previous findings suggest that anxiety is affected by dietary oils that contain unsaturated fatty acids. However, few studies have compared dietary oils containing saturated and unsaturated fatty acids with regard to their effects on emotional behaviors using the same experimental model.

Some evidence suggests physiological effects of fatty acids. Ketone bodies, which re-formed on consumption of ketogenic diets through cellular metabolism, enhance gamma-aminobutyric acid (GABA) synthesis, decrease neuron membrane potentials, and have antioxidant effects [9]. *In vitro* recording of pyramidal neurons in the rat cerebrum indicated that DHA potentiated the *N*-methyl-*D*-apartic acid (NMDA) response in the presence of glycine, whereas oreic acid caused a slight potentiation and polysaturated fatty acids had no effect [10]. Therefore, the altered emotional memory observed in anxiety via functions of GABA and NMDA receptors likely also depends on the species of fatty acids present.

We previously screened epilepsy-dependent molecules using a temporal lobe epilepsy (TLE) [11, 12] mouse model in which kindling stimulation is applied repeatedly to the amygdala [13] and found that sialyltransferase ST3Gal IV is upregulated in neurons located within the neural circuits that kindling stimulation propagates [14]. To determine whether ST3Gal IV is a determinant of seizure occurrence, we established the ST3Gal IV-knockout (KO) mouse model. Then, we demonstrated that ST3Gal IV-KO mice failed to show seizures with repeated kindling stimulation. Additionally, the ST3Gal IV-KO mice exhibited decreased exploration/acclimation in the open field, enhanced cognitive fear in delay auditory fear conditioning, and increased immobility in the forced swim test [15]. These findings indicate that ST3Gal IV-KO mice are a novel experimental model to understand the molecular mechanisms underlying epilepsy and anxiety-related behaviors.

The kindling stimulation also increased growth hormone (GH) levels in neuronal cells during development of epilepsy and following acquisition of seizures, and infusion of GH into the hippocampus markedly enhanced the progression of epileptogenesis [16]. In contrast, ST3Gal IV-KO mice that exhibit no epileptic seizures show down-regulation of GH and Igf1 mRNA in the cerebral cortex [15]. Additionally, it was shown that hippocampal GH is upregulated during memory formation [17] in first showing that GH is expressed in the brain and not transported from the pituitary gland and locally GH mRNA is expressed in pyramidal neurons of the CA3-subfield of the hippocampus [16]. Further investigations showed that subcutaneous injection of GH altered the expression of GH receptor and NMDA receptor mRNAs in the hippocampus, depending on the ages of rats [18]. In rat hippocampal slices *in vitro*, application of GH enhanced AMPA and NMDA receptor expression and induced excitatory post-synaptic potentials [19]. These findings suggest a physiological relationship between GH and fatty acids might mediate via the NMDA receptor in the hippocampus, as growth hormone and Igf1 are known to be principal hormones involved in lipid metabolism [20]. Given the differences in brain lipid metabolism between ST3Gal IV-KO and wild-type (WT) mice, it is suspected that emotional behaviors that are affected by dietary triglycerides will differ between ST3Gal IV-KO and WT mice. Such a difference would suggest that the ST3Gal IV-KO model mouse is useful to analyze the molecular mechanisms underlying the physiological effects of fatty acids obtained from the diet in the brain. The present study investigated which and how dietary triglycerides (oils), including saturated and unsaturated fatty acids, affected emotional behaviors of ST3Gal IV-KO and the littermate WT mice.

## Materials and Methods

### Ethics statement

This study was carried out in accordance with the *Guidelines for Proper Conduct of Animal Experiments* published by the Science Council of Japan (2006), and all efforts were made to minimize suffering and the number of animals used. The protocol was approved by the Committee on the Ethics of Animal Experiments of Kyoto Sangyo University (Approval No. 2010–28 and 2012–08).

### Animals

ST3Gal IV gene-deficient mice were generated previously [15] and backcrossed for 12 to 14 generations with C57Bl/6J mice. Mice were housed in a temperature-controlled room (22–24°C) with a 12-h light/dark cycle (lights on at 0800) and were provided free access to food and water. Four mice were bred in open-top plastic cages [225 mm (width) × 338 mm (length) × 140 mm (height)]. The cage tops were covered with stainless steel wire grid lids, and the cage floors were covered with sawdust that was changed weekly. All behavioral experiments (open field, fear conditioning, and forced swim tests) were performed between 0800 and 1200.

### Oil-rich diets

Experimental group mice were fed a diet that included one of the following combinations of dietary oils: (1) 20% soybean oil including 10.6% n-6 unsaturated fatty acid (linoleic acid); (2) 18% fish oil including 6.3% n-3 polyunsaturated fatty acids (DHA and EPA), plus 2% soybean oil; and (3) 18% POP-SOS triglycerides including 13.4% saturated fatty acids, plus 2% soybean oil. Control group mice were fed a control diet consisting of AIN93G (S1 Table). The diets were adjusted to contain the same number of calories per unit of weight by changing the amount of β cornstarch and cellulose powder used in the pellets. Although AIN93G generally includes 7% soybean oil, all diets contained 2% soybean oil to ensure that mice received the essential fatty acids necessary for adequate nutrition and development.

Feed powders were mixed as described in S1 Table with distilled water. The resulting pastes were shaped into 13.5 × 24.5 × 10-mm rectangles, freeze-dried under N_2_ gas, and stored at -80°C in screw cap bottles. The fresh feed stored at -80°C was thawed and given to mice. Feed that remained within the bait box for 2 days was discarded and replaced with fresh feed. Mice were fed the prepared diets from postnatal day 24 until the end of the experiments.

### Behavioral tests

Mice were subjected to 3 behavioral tests from 9 weeks of age. Activity, exploration, and acclimation were evaluated with the open field test. Anxiety behaviors were evaluated with the auditory fear conditioning test, and depressive behaviors were evaluated with the forced swim test. These tests were performed sequentially at 1-week intervals.

#### Open field test

The open field test was performed by placing mice in a square-shaped arena (490 mm wide × 490 mm long × 195 mm high) that had a ceiling height 125 cm above the field floor. A mouse was placed in the field for 5 min and then returned to the home cage for 5 min, and this procedure was performed twice. The light intensity of the fluorescent lamp that lit the open field was set to 350 lux. Data were collected with a camera placed 470 mm above the field floor and analyzed automatically with TimeOFCR4 (O’hara & Co., Ltd.).

#### Fear conditioning test

Auditory fear conditioning was performed by placing mice in a clear, square-shaped arena (170 mm wide × 100 mm long × 100 mm high) that was fitted with a metal grid shock floor through which scrambled footshocks were delivered as unconditioned stimuli (US; 0.3 mA, 1 s; O’hara & Co., Ltd.). The arena was placed into an acoustic isolation box (background noise level 50 dB, 200 lux), and sounds were delivered as conditioning stimuli (CS; white noise, 65 dB, 10 s) through a speaker positioned on the right side of the chamber. On the day of training, each mouse was placed in the clear square-shaped arena and allowed to freely explore for 1 min. A 65-dB CS was then presented for 10 s, and a 0.3-mA footshock was delivered during the final 1 s of tone presentation. The CS and US were delivered twice with an interval of 20 s, which were delivered automatically by the tone generator and shock controller. Mice were placed in the arena for 3 min and then returned to their home cages. Twenty-four hours later, the contextual fear conditioning test was performed in the acoustic isolation box (200 lux). Mice were placed in the same clear square-shaped arena for 5 min, but the CS or US was not delivered. Twenty-four hours after the contextual fear conditioning test, mice were subjected to a cued test in which they were placed in a solid, gray, square-shaped arena within an acoustic isolation box (background noise level 55 dB and 50 lux) and allowed to explore freely for 1 min. After the first exploration period, the CS (tone) was presented for 1 min, and animals were again allowed to explore for 2 min. All data were collected and analyzed automatically with Time FZ1 (O’hara & Co., Ltd.), and the percentage of total freezing time was calculated. Freezing behavior was defined as periods longer than 2 s during which the animal remained motionless.

#### Forced swim test

A clear tank (11.3-cm diameter, 22.3-cm height) was filled with water (24°C) to a depth of 15 cm, which was replenished with fresh water between trials. The tank was placed in a box that was lit with a 1010-lux incandescent lamp. Each trial was 6 min long, and data were collected and analyzed automatically with Time FZ1.

### Statistics

The data are presented as mean ± SEM (standard error of the mean); p < 0.05 was considered statistically significant. The data were analyzed with a one-way ANOVA (analysis of variance) using Excel (Microsoft) and Prism 6 (GraphPad Software, Inc.). The combination of genotype and food was considered the independent factor. Statistically significant effects were further evaluated using Tukey’s multiple comparison test to compares every mean with every other mean, when the one-way ANOVA was significant at p < 0.05 level. On the other hand, when two means were compared, the comparison is affected by the effects of other means and the significance is typically more difficult to calculate than significance obtained using the unpaired *t*-test for comparison between two groups. Therefore, the data were analyzed with unpaired *t*-tests also for comparisons between ST3Gal IV-KO and WT mice ($ and $$). Additionally, the data were analyzed with unpaired *t*-tests for comparisons between the first and second entries or between Day 1 and Day 2 (#). The number of asterisks for the p values indicates the following significance levels: ****p < 0.0001, ***p < 0.0005, **p < 0.005, and *p < 0.05.

## Results

### Open field test

ST3Gal IV-KO and WT mice that were fed an oil or control diet for 70 days were subjected to the open field test. The total distance travelled and the frequency of entry into the center area (which induces mild anxiety) were used to quantify activity, exploration, and acclimation.

**Fig 1:**
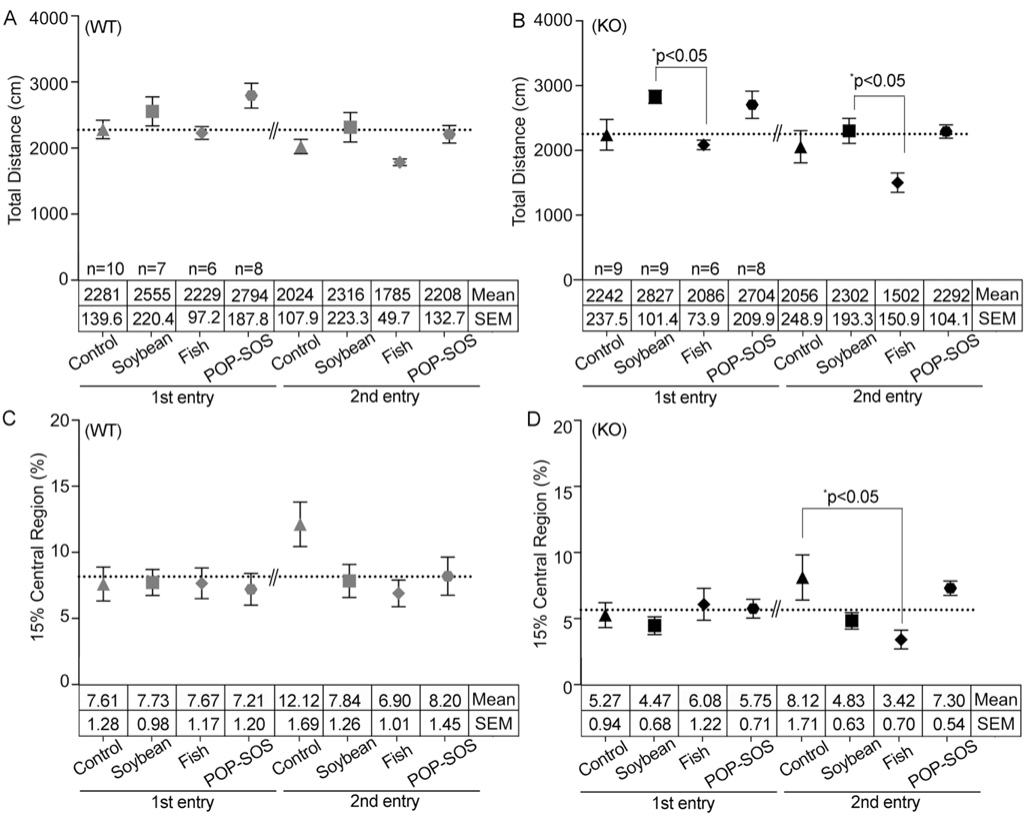
Differential effects of oil-rich diets on mouse ambulation in the open field test. Total distance (cm) denotes the distance traveled two times during the 5 min in the arena (wild-type mice (WT) in A and ST3Gal IV-KO mice (KO) in B); 15% central region (%) denotes time per 5 min that mice entered and stood in the 15% center area (C and D). The x-axis shows the type of diet as listed in S1 Table: Soybean, soybean oil diet; Control, AIN93G; Fish, fish oil diet; POP-SOS, POP-SOS diet. Data represent the mean ± SEM in the horizontal tables below each point, and the numbers of mice are presented in the horizontal table in A (WT) and B (KO). Statistical analysis was performed by ordinary one-way ANOVA with Tukey’s multiple comparison test to compare the oil types: F(3, 27) = 2.376, p = 0.092 in the first entry; F(3, 27) = 2.294, p = 0.101 in the second entry; dotted line (average) = 2274 in A (WT): F(3, 28) = 3.663, *p = 0.024, Tukey, *p = 0.050 [soybean oil-KO with fish oil-KO] in the first entry; F(3, 28) = 3.112, *p = 0.042, Tukey, *p = 0.046 [soybean oil-KO with fish oil-KO] in the second entry; dotted line = 2251 in B (KO): F(3, 27) = 0.036, p = 0.991 in the first entry; F(3,27) = 2.637, p = 0.070 in the second entry; dotted line = 8.160 in C (WT): F(3, 28) = 0.638, p = 0.597 in the first entry; F(3, 28) = 3.652, *p = 0.024, Tukey, *p = 0.037 [fish oil-KO with control-KO] in the second entry; dotted line = 5.655 in D (KO). Graphs presented *p* values of Tukey’s test that indicate the significant levels. Additionally, statistical values obtained by un-paired *t*-test are described in S1 Fig, in which $ shows statistical significance between WT and KO mice that were fed any type of oil-rich diet (red) and ^#^hashes show significant differences between the first and second entries for the ST3Gal IV-KO and WT mice.

As described previously [15], there was no difference in total distance travelled between ST3Gal IV-KO and WT mice fed the control diet (S1 Fig A and B). In addition, the total distance traveled tended to be shorter for the second entry compared to the first entry (# in S1 Fig B), a finding that suggests the influence of acclimation to the field. A fish oil diet decreased the distance travelled by ST3Gal IV-KO mice on the second entry by 0.7-fold compared to that on the first entry (unpaired *t*-test, F(5, 5) = 4.17, ^#^p = 0.006 in S1 Fig B). Tukey’s comparison test at both the first and second entries indicated that ST3Gal IV-KO mice fed the soybean and fish oil diets traveled the longest and the shortest total distances (Tukey, *p = 0.050 and *p = 0.046), respectively (Fig. 1B). This was confirmed by comparisons in the moving speed of the mice (Tukey, **p = 0.007 in S2 Fig). These findings suggest that activity is increased by a soybean oil diet in ST3Gal IV-KO mice, but activity is decreased in ST3Gal IV-KO mice by a fish oil diet (Table 1).

**Table 1:** Dietary oils that affected the behaviors of ST3Gal IV-KO and WT mice.

	Tests in which KO mice did not show symptoms		Tests in which KO mice showed symptoms		
	Activity	Context fear	15% Center	Tone fear	Forced swim
Improvement in KO mice	*Soybean oil*	No	No	**POP-SOS (0.4-fold** ^**#**^ **)**	No
				*Soybean oil*, *Fish oil*	
Aggravation in KO mice	*Fish oil*	**Fish oil (1.4-fold** ^**#**^ **)**	**Fish oil(2.4-fold** ^**#**^ **)**	No	**Soybean oil (1.7-fold** ^**#**^ **)**
Improvement in WT mice	No	**POP-SOS (0.6-fold** ^**#**^ **)**	No	No	No
		**Soybean oil (0.5-fold** ^**#**^ **)**			
Aggravation in WT mice	No	*Fish oil*	No	No	No

This table shows the effect of triglycerides (oils) in each behavioral test, separated according to whether ST3Gal IV-KO mice fed the control diet showed symptoms.

**Fig 2:**
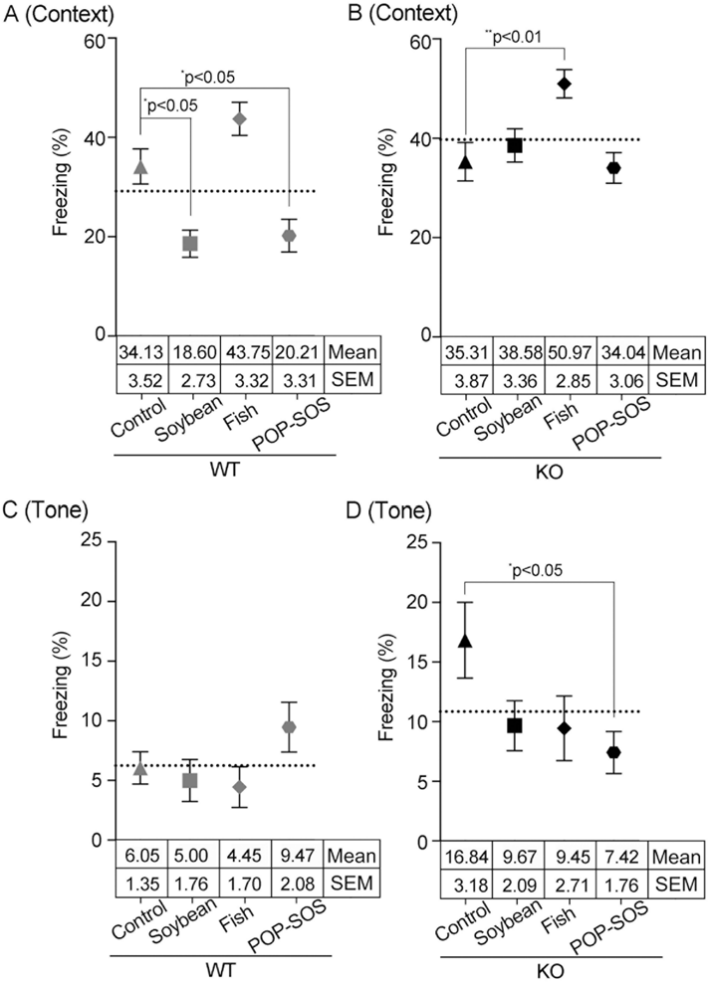
Effects of oil-rich diet on mouse freezing behavior during the auditory fear conditioning test. Mice underwent contextual fear testing 1 day following the first fear conditioning training (S3 Fig) and the last 3 min of each trial was analyzed for a period of 5 min (A and B). Mice underwent the tone fear test 2 days following the first fear conditioning training and the response of the mice was analyzed in the last 2 min for a period of 4 min (C and D). Graphs were separated between WT and ST3Gal IV-KO (KO) mice. The vertical line shows the mean ± SEM of the % total freezing time per 30 s and the numbers of mice used were the same as those presented in the horizontal table in Fig. 1A (WT) and B (KO). Statistical analysis was performed by ordinary one-way ANOVA with Tukey’s multiple comparison test to compare oil types: F(3, 196) = 11.56, ****p<0.0001; Tukey, *p = 0.022 [control-WT with soybean oil-WT], ****p<0.0001 [fish oil-WT with soybean oil-WT], *p = 0.010 [POP:SOS-WT with control-WT], ****p<0.0001 [POP:SOS-WT with fish oil-WT]; dotted line (average) = 29.173 in A (WT): F (3, 196) = 5.526, **p = 0.0012; Tukey, **p = 0.007 [fish oil-KO with control-KO], **P = 0.001 [POP:SOS-KO with fish oil-KO]; dotted line = 39.725 in B (KO): F (3, 121) = 1.697, p = 0.171; dotted line = 6.240 in C (WT): F (3, 121) = 2.911, *p = 0.037; Tukey, *p = 0.026 [POP:SOS-KO with control-KO]; dotted line = 10.845 in D (KO). Each graph shows only significance of differences determined by Tukey’s test for comparison of each type of oil diet with the control diet.

**Fig 3:**
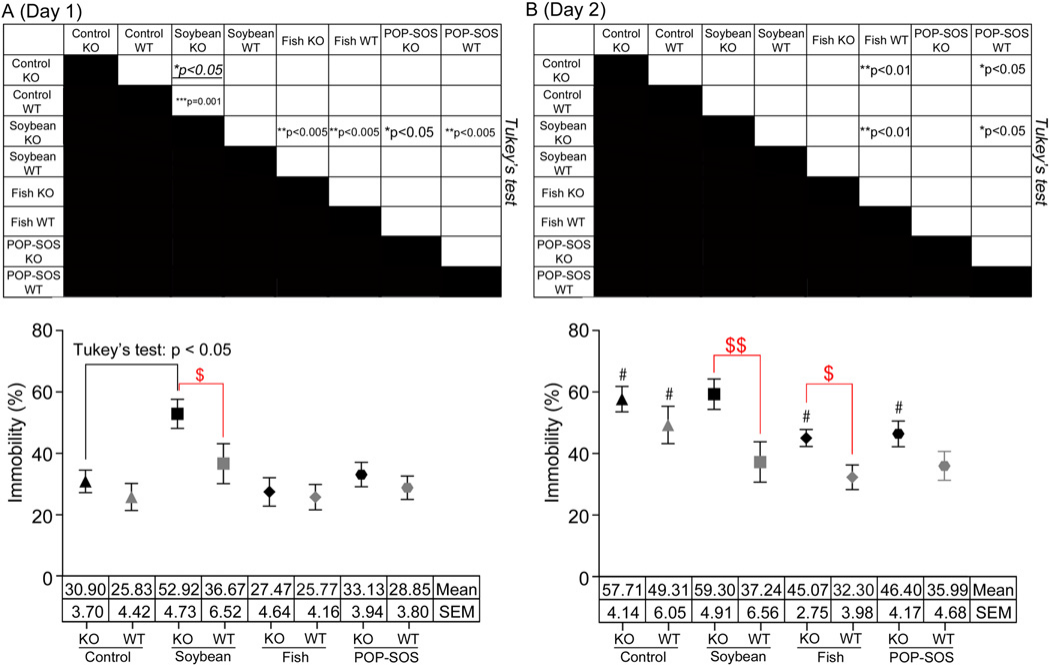
Differential effects of oil-rich diets on mouse immobility in the forced swim test. Mice entered the forced swim pool arena once a day for 2 days (day 1 in A and day 2 in B). Trials were 6 min long and the last 4 min of data were analyzed. The vertical line shows the mean ± SEM of the % total immobile time per 30 s, and the numbers of mice used were the same as those shown on the horizontal table in Fig. 1A and B. Significant difference by Tukey’s multiple comparison test is shown in the tables presented above the figures. Significant values obtained by the two-tailed unpaired *t*-test are described below, and values showing no difference by unpaired *t*-test are presented in S1 File. In A, ANOVA: F(7, 192) = 3.56, **p = 0.001; two-tailed unpaired *t*-test, in which $ shows statistical significance between WT and KO mice (red): F(15, 19) = 1.52, ^$^p = 0.047 for soybean oil diet. In B, ANOVA: F(7, 192) = 4.04, ***p = 0.0004; two-tailed unpaired *t*-test (red): F(15, 19) = 1.43, ^$$^p = 0.010 for soybean oil diet; F(23, 23) = 2.10, ^$^p = 0.011 for fish oil diet. ^#^Hashes denote statistical significance by two-tailed unpaired *t*-test between the first and second days (B). F(23, 23) = 1.26, ^#^p < 0.0001 in ST3Gal IV-KO with control diet; F(27, 27) = 1.87, ^#^p = 0.003 in WT with control diet; F(23, 23) = 2.86, ^#^p = 0.002 in ST3Gal IV-KO with fish oil diet; F(31, 31) = 1.120, ^#^p = 0.024 in ST3Gal IV-KO with POP-SOS diet.

Activity and 15% center results in the open field test (Fig. 1), results for the context and tone fear tests (Fig. 2), and results for the forced swim test (Fig. 3) are summarized.

Soybean oil: diet including 20% soybean oil; Fish oil: diet including 18% fish oil and 2% soybean oil; POP-SOS: diet including 18% POP-SOS and 2% soybean oil.

^#^Hash shows an increased difference between the oil diet group and control diet group, with significance confirmed using Tukey’s comparison test after one-way ANOVA.

Italic shows tendency of differential effect, when behaviors in mice fed a triglyceride (oil)-rich diet were compared with those in mice fed a control diet (AIN93G) for ST3Gal IV-KO and WT mice. No: no effect of triglycerides (oils) on behavior.

There were no differences in the frequency of center field entries on the first entry between ST3Gal IV-KO and WT mice fed diets other than soybean oil (unpaired *t*-test, F(6, 8) = 1.64, ^$^p = 0.013 in S1 Fig C). On the other hand, WT mice fed the control diet showed a 1.6-fold increase in center field exploration on the second entry (unpaired *t*-test, F(9, 9) = 1.74, ^#^p = 0.047 in S1 Fig D); however, ST3Gal IV-KO mice failed to show such an increase, which is consistent with previous findings [15]. This indicates that ST3Gal IV-KO mice acclimate poorly to their environment. In WT mice, center access at the second entry was decreased 0.6-fold with a soybean oil or fish oil diet and by 0.7-fold with a POP-SOS diet as compared to the control diet (Fig. 1C), however, there was little significant difference (one-way ANOVA, F(3, 27) = 2.64, p = 0.070 in Fig. 1C). It suggests the possibility that oil-containing diets decrease exploration and acclimation in WT mice. ST3Gal IV-KO mice fed the fish oil diet showed a 0.4-fold decrease in center access at the second entry compared to mice fed the control diet (Tukey, *p = 0.037 in Fig. 1D). This finding suggests that the fish oil diet makes it harder to acclimate ST3Gal IV-KO mice. Tukey’s test among all groups shown in Fig. 1 showed only a difference between the fish oil diet and control diet in ST3Gal IV-KO mice in center access of the open field tests; however, a little significant difference may have resulted from the small sample size.

### Fear conditioning test

ST3Gal IV-KO and WT mice that were fed an oil or control diet for 80 days were subjected to an auditory fear conditioning test. Mice received context and tone tests 24 h and 48 h following the conditioning, respectively. On the first day of conditioning training, a slight difference was observed between ST3Gal IV-KO and WT mice that ate any of the oil diets (one-way ANOVA; F(7, 142) = 2.11; *p = 0.047), with no significance in Tukey’s test (S3 Fig). On the second day of testing, the behavior of the mice was examined in the same experimental chamber, but in the absence of the CS or US (Fig. 2A and B, and S4 Fig A). There was no difference between ST3Gal IV-KO and WT mice in the control diet condition (S4 Fig A), as described previously [15]. In WT mice, the POP-SOS diet decreased the freezing percentage by 0.6-fold relative to mice fed the control diet (Tukey, *p = 0.010), and the soybean oil diet decreased the freezing percentage by 0.5-fold relative to mice fed the control diet (Tukey, **p = 0.022) (Fig. 2A). This indicates that a POP-SOS and soybean oil diet alleviated contextual fear in WT mice. Furthermore, a fish oil diet feeding enhanced the freezing percentages of ST3Gal IV-KO mice to 1.4-fold (Tukey, **p = 0.007) relative to the control diet, and that of WT mice by 2.4- and 2.2-fold (Tukey, ****p < 0.0001) relative to the soybean oil and POP-SOS diets, respectively, indicating a strengthening of contextual fear responses.

On the third day of our experimental battery, tone testing was examined in a novel environmental field without a US (Fig. 2C and D, and S4 Fig B). ST3Gal IV-KO mice fed the control diet showed a tremendous increase (2.8-fold) in freezing behavior compared to WT mice, as described previously (Tukey, **p = 0.009, S4 Fig B) [15]. There were no differences in freezing levels among WT mice under all diets, showing that the present oils do not affect tone fear of normal mice (Fig. 2C). However, ST3Gal IV-KO mice showed that the POP-SOS diet decreased freezing percentages to the baseline level (0.4-fold) observed in WT mice (Tukey, *p = 0.026 in Fig. 2D), suggesting that the POP-SOS diet completely rescued ST3Gal IV-KO mice from tone-induced fear. Furthermore, soybean and fish oil diets diminished the freezing percentage of ST3Gal IV-KO mice by 0.6-fold (Tukey, p = 0.219 and p = 0.162, respectively). These findings suggest remarkable differential effects of oil-rich diets on context and tone fear in ST3Gal IV-KO and WT mice, respectively.

### Forced swim

ST3Gal IV-KO and WT mice that were fed an oil or control diet for 90 days underwent forced swim tests once a day for 2 days. Just as repeated exposure to stress is the main factor that triggers depressive symptoms in humans [21], the forced swim test is a widely utilized animal model of “behavioral despair.” Normal mice show increased immobility on the second day of testing as compared to the first day [22]. In the present study, both WT and KO mice fed the control diet also showed a 1.9-fold increased degree of immobility on day 2 (unpaired *t*-test, F(27, 27) = 1.87, ^#^p = 0.003 in WT and F(23, 23) = 1.26, ^#^p < 0.0001 in ST3Gal IV-KO mice in Fig. 3B and S1 File). ST3Gal IV-KO mice showed that a soybean oil diet induced greater immobility by 1.7-fold that of the control diet on the first day of testing (Tukey, *p = 0.024, Fig. 3A) and maintained the same level of immobility on the second day. This result suggests that the soybean diet strengthened depressive behavior in ST3Gal IV-KO mice.

## Discussion

The present study used a control diet (AIN93G) and 3 types of diets containing 20% oil, including fish oil, soybean oil, and POP-SOS triglycerides (S1 Table). It then was evaluated whether the oil-rich diets differentially influence animals’ responses to mild anxiety and stimulus-evoked stress, as summarized in Table 1. In particular, Tukey’s comparison test indicates that (1) WT mice that were fed POP-SOS- and soybean oil-rich diet showed decreased contextual fear; (2) ST3Gal IV-KO mice that were fed POP:SOS-rich diet showed decreased tone fear; (3) ST3Gal IV-KO mice that were fed fish oil- and soybean oil-rich diet showed aggravated contextual fear and “swimming despair,” respectively.

First, oil-rich diets may function on each neural pathway to change anxiety- and depression-like behaviors. For example, WT mice experienced rescue from contextual fear responses on consuming POP-SOS-rich diets (bold in column 3, Table 1, Fig. 2A), whereas WT mice showed no difference in tone fear responses on consumption of any of the diets in the present study (column 5, Table 1, Fig. 2C). Contextual stimuli are processed in the hippocampus, and hippocampal afferents transmit information to neurons of the basolateral and basomedial nuclei in the amygdala [23]. The basolateral and basomedial divisions connect with the central nuclei within the amygdala and then finally project out of the amygdaloid nuclei to the brainstem areas controlling fear responses [24, 25]. Tone stimuli are conveyed by afferents relaying auditory information from the medial geniculate nucleus of the thalamus to the lateral amygdala nucleus [24]. Additionally, sensory information from the auditory cortex is transmitted to these same nuclei of the amygdala [26]. These suggest the neural pathways involved in context and tone fear are differentially affected by the oil diet.

Second, oil-rich diets that affect behavior were different between ST3Gal IV-KO and WT mice. For example, the POP-SOS diet improved tone fear in ST3Gal IV-KO mice and context fear in WT mice (columns 3 and 5 in Table 1). On the other hand, the soybean oil diet containing 10.6% linoleic acid (an n-6 fatty acid) aggravated “swimming despair” only in ST3Gal IV-KO mice (column 6 in Table 1) but improved context fear in WT mice. Thus, ST3Gal IV-KO mice may be different from WT mice in brain lipid metabolism via dietary oils containing n-6 fatty acids.

Third, the total distance (Tukey, *p = 0.046, comparison with soybean oil) and duration of 15% center (Tukey, *p = 0.037, comparison with control) decreased in ST3Gal IV-KO mice that were fed a fish diet in the open field test (the second entry in Fig. 1B and D). This indicates that the fish oil-rich diet reduced activity and exploration in ST3Gal IV-KO mice (columns 2 and 4 in Table 1), which was consistent with the previous finding that n-3 fatty acid-deficient diets increase the locomotor activity of rodents [4]. Further, the fish oil diet resulted in a severe increase in contextual fear in WT (Tukey, ****p < 0.0001, comparisons with soybean oil and POP-SOS diets) and ST3Gal IV-KO (Tukey, **p = 0.007, comparison with control diet) mice (Fig. 2A and B). These results suggest that dietary intake of excess fish oil increases cognitive anxiety in mice.

A novel point of the present study is that the POP-SOS diet improved mouse symptoms of anxiety and depression, such as contextual fear in WT mice (Tukey, *p = 0.010) and tone fear in ST3Gal IV-KO mice (Tukey, *p = 0.026) (Fig. 2). It has been reported that saturated fatty acids including palmitic acid and stearic acid are associated with an increased risk of coronary heart disease and some tumors [27], and that saturated fatty acids produce Alzheimer’s-like hyperphosphorylation with the brain in *in vitro* assays [28]. However, the POP-SOS diet in the present study alleviated mouse anxiety and depression symptoms in the presence of a stressor. Thus far, little is known about the effects of triglycerides, including the effects of saturated fatty acids on emotional behaviors. The present study shows that a mixture of palmitic acid, stearic acid, and oleic acid is suitable for alleviating anxiety symptoms in a rodent model, and represents an important advance in understanding the neural effects of dietary triglycerides.

The present study focused on observations of emotional and cognitive behaviors in mice that were fed different types of oil, and not on the mechanistic relationships or differences in physiology. However, previous *in vivo* recordings in the dentate gyrus of the hippocampus indicated reduced long-term potentiation in rats fed a ketogenic diet including saturated fatty acids for 3 weeks [29], whereas pups delivered from a dam fed DHA during pregnancy and lactation showed elevation [30]. These findings are consistent with the reduction of context fear in WT mice that were fed the POP-SOS and soybean diets and also similar to the high tendency of fear in WT and ST3Gal IV-KO mice that were fed the fish oil diet including DHA in the present study (column 3 in Table 1). These effects may be caused by the adequate potentiation of the NMDA response by application of DHA but not the polysaturated fatty acid or oreic acid included in POP-SOS [10]. Behavioral evidence also indicates that infusion of NMDA receptor antagonist (AP7) into the hippocampus results in attenuation of the context fear [31]. In the present study, fish oil diet including DHA may enhance the context fear via NMDA receptor in the hippocampus. Another behavioral evidence indicates that loss of GABA receptor that highly expresses specifically in the hippocampus enhances the context fear [32]. As GABA synthesis increases by ketone bodies [9], which re-formed from triglyceride including palmitic aicd and stearic acid, in the present study, increase of GABA synthesis by POP-SOS may result in reduction of the context fear via GABA receptor in the hippocampus.

Additionally, GH is expressed in pyramidal neurons of the CA3 subfield of the hippocampus [16], and a previous *in vitro* rat hippocampal slice study showed that application of GH enhanced AMPA and NMDA receptors-mediated excitatory post-synaptic potentials [19]. Thus, the effect of GH on NMDA receptors appears to be similar to that of DHA [10]. In contrast, ST3Gal IV-KO mice showing low expression of GH exhibited little difference in context fear among the control, POP-SOS, and soybean oil diet groups, which was different from what was observed in WT mice fed the POP-SOS and soybean oil diets. As increased GH levels result in a marked increase in lipolysis and free fatty acid levels [33], the decrease of brain GH levels in ST3Gal IV-KO mice may inhibit the degradation of POP-SOS triglycerides, thus resulting in a lack of effect for context fear, even though the mice were fed the POP-SOS diet. However, brain GH may have no effect on the degradation of fish oil including DHA, as ST3Gal IV-KO mice fed the fish oil diet still showed elevated context fear similar to that in WT mice. Thus, analyses of brain lipid content in the present mice may provide insights into the lipid metabolism mediated by brain GH, which is associated with emotional and cognitive behaviors.

Finally, the present study demonstrates that emotional behaviors are differentially influenced by the type and quantity of dietary oils (triglycerides), which contain different fatty acids. Furthermore, the differential effects of the different dietary oils were observed in the open field, fear conditioning, and forced swim tests. It was suggested that different lipid metabolism might exist in neural regions responsible for each behavior. It remains unclear how digestion, adsorption, and oil resynthesis are involved in brain lipid metabolism. Nevertheless, the present findings at least suggest that the ST3Gal IV-KO mouse is a viable model for investigating the role of brain lipid metabolism in modulating anxiety- and depression-like behaviors. The present study will open a path for further analyses of the involvement of triglycerides (oils) in emotional and cognitive behaviors.

## Supporting Information

S1 Fig Effects of oil-rich diet on mouse ambulation in the open field test. Graphs of total distance (cm) (A, B) and 15% central region (%) (C, D) shown in Fig. 1 were separated between the first entry (A, C) and second entry (B, D). Statistical values of the two-tailed unpaired *t*-test evaluated difference between WT and KO mice that fed the same diet. There was little difference in total distance in A and B. In C, the two-tailed unpaired *t*-test, in which $ shows statistical significance between WT and KO mice (red): F(9, 8) = 2.05, p = 0.17 in control; F(6, 8) = 1.64, ^$^p = 0.013 in soybean; F(5, 5) = 1.09, p = 0.37 in fish; F(7, 7) = 2.89, p = 0.31 in POP-SOS. In D: F(9, 8) = 1.08, p = 0.12 in control; F(6, 8) = 3.08, ^$^p = 0.038 in soybean; F(5, 5) = 2.05, ^$^p = 0.018 in fish; F(7, 7) = 7.10, p = 0.57 in POP-SOS. ^#^Hashes denote statistical significance obtained using the two-tailed unpaired *t*-test between the first and second entries. Total distance in Fig. 1A and B: F(8, 8) = 1.10, p = 0.60 in control-KO; F(9, 9) = 1.67, p = 0.16 in control-WT; F(8, 8) = 3.64, ^#^p = 0.029 in soybean-KO; F(6, 6) = 1.03, p = 0.46 in soybean-WT; F(5, 5) = 4.17, ^#^p = 0.006 in fish-KO; F(5, 5) = 3.82, ^#^p = 0.002 in fish-WT; F(7, 7) = 4.07, p = 0.10 in POP-SOS-KO; F(7, 7) = 2.00, ^#^p = 0.023 in POP-SOS-WT. 15% central region in C and D: F(8, 8) = 3.29, p = 0.16 in control-KO; F(9, 9) = 1.74, ^#^p = 0.047 in control-WT; F(8, 8) = 1.14, p = 0.70 in soybean-KO; F(6, 6) = 1.65, p = 0.94 in soybean-WT; F(5, 5) = 2.98, p = 0.09 in fish-KO; F(5, 5) = 1.33, p = 0.63 in fish-WT; F(7, 7) = 1.68, p = 0.10 in POP-SOS-KO; F(7, 7) = 1.46, p = 0.61 in POP-SOS-WT. (TIF)
(TIF)

S2 Fig Effects of oil-rich diet on moving speed (cm/s) of mice in the open field test. Moving speed (cm/s) at the first entry was extracted from Fig. 1. Statistical analysis was performed by ordinary one-way ANOVA with Tukey’s multiple comparison test for comparison of the 2 genotypes that ate foods containing each type of oils: F(7, 55) = 3.59, **p = 0.003; Tukey’s: **p = 0.007 [fish-KO with soybean-KO], *p = 0.049 [fish-WT with soybean-KO], and *p = 0.035 [fish-KO with POP-SOS-WT]. (TIF)
(TIF)

S3 Fig Effects of oil-rich diet on the first fear conditioning training of mice. Mice received training stimuli coupled with tone and electrical shock twice in a chamber where the mouse was resting on day 1. Trials were 3 min in length and the last 1.5 min of each trial was analyzed. Statistical analysis was performed by ordinary one-way ANOVA with Tukey’s multiple comparison tests to compare the 2 genotypes that ate foods containing each type of oils: F(7, 142) = 2.11; *p = 0.047, no significance in Tukey’s. (TIF)
(TIF)

S4 Fig Effects of oil-rich diet on contextual and tone fear tests. Graphs of context freezing (%) (A) and tone freezing (%) (B) shown in Fig. 2 were gathered. Statistical analysis was performed by ordinary one-way ANOVA to compare the 2 genotypes on consumption of food containing each type of oil: F(7, 392) = 9.84, ****p < 0.0001 in A; F(7, 242) = 3.12, **p = 0.004 in B. Significant difference by Tukey’s multiple comparison test is shown in the tables presented above the figures. The *p* value for Tukey’s test in mice that ate any oil-rich diet compared to mice that ate a control diet is underlined and written in bold font. Differences identified with Tukey’s test between any oil-rich diet and the control diet are shown with a large-shaped line (ST3Gal IV-KO mice) and a large dotted line (WT mice). Significant difference by Tukey’s test between ST3Gal IV-KO and WT mice that ate the same diet is denoted with a red line. (TIF)
(TIF)

S1 File Effects of oil-rich diet on contextual and tone fear tests. (DOCX)

S1 Table Composition of diets. (DOCX)

S2 Table Fatty acid composition in brain and dietary oil. (DOCX)
