# Supplementary figures and images for: Differential Effects of Dietary Oils on Emotional and Cognitive Behaviors
Source: PLoS One. 2015 Mar 23;10(3):e0120753. doi: 10.1371/journal.pone.0120753 (PMC4370753; doi:10.1371/journal.pone.0120753)

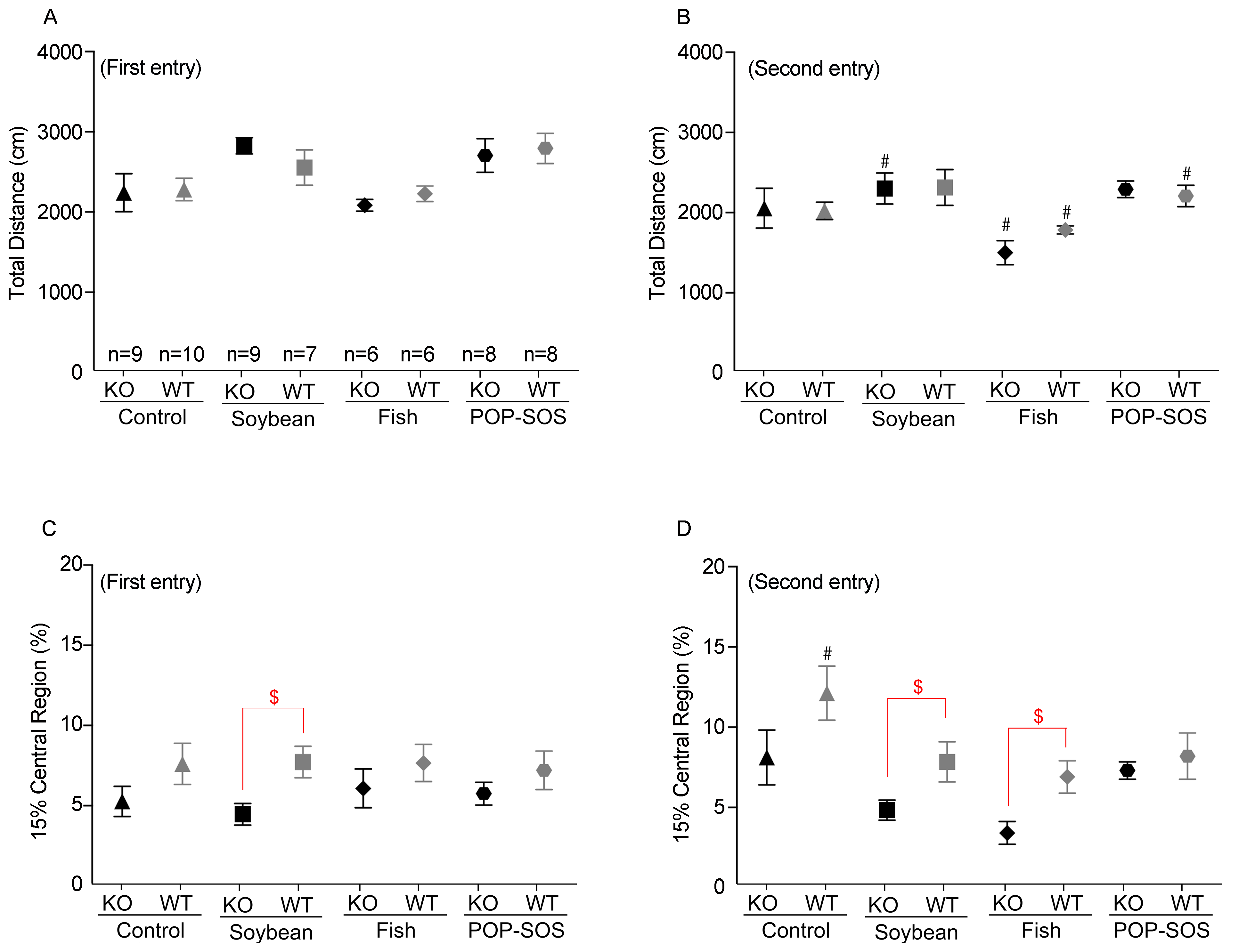

Supplement: S1 Fig — Graphs of total distance (cm) (A, B) and 15% central region (%) (C, D) shown in Fig. 1 were separated between the first entry (A, C) and second entry (B, D). Statistical values of the two-tailed unpaired t-test evaluated difference between WT and KO mice that fed the same diet. There was little difference in total distance in A and B. In C, the two-tailed unpaired t-test, in which $ shows statistical significance between WT and KO mice (red): F(9, 8) = 2.05, p = 0.17 in control; F(6, 8) = 1.64, $p = 0.013 in soybean; F(5, 5) = 1.09, p = 0.37 in fish; F(7, 7) = 2.89, p = 0.31 in POP-SOS. In D: F(9, 8) = 1.08, p = 0.12 in control; F(6, 8) = 3.08, $p = 0.038 in soybean; F(5, 5) = 2.05, $p = 0.018 in fish; F(7, 7) = 7.10, p = 0.57 in POP-SOS. #Hashes denote statistical significance obtained using the two-tailed unpaired t-test between the first and second entries. Total distance in Fig. 1A and B: F(8, 8) = 1.10, p = 0.60 in control-KO; F(9, 9) = 1.67, p = 0.16 in control-WT; F(8, 8) = 3.64, #p = 0.029 in soybean-KO; F(6, 6) = 1.03, p = 0.46 in soybean-WT; F(5, 5) = 4.17, #p = 0.006 in fish-KO; F(5, 5) = 3.82, #p = 0.002 in fish-WT; F(7, 7) = 4.07, p = 0.10 in POP-SOS-KO; F(7, 7) = 2.00, #p = 0.023 in POP-SOS-WT. 15% central region in C and D: F(8, 8) = 3.29, p = 0.16 in control-KO; F(9, 9) = 1.74, #p = 0.047 in control-WT; F(8, 8) = 1.14, p = 0.70 in soybean-KO; F(6, 6) = 1.65, p = 0.94 in soybean-WT; F(5, 5) = 2.98, p = 0.09 in fish-KO; F(5, 5) = 1.33, p = 0.63 in fish-WT; F(7, 7) = 1.68, p = 0.10 in POP-SOS-KO; F(7, 7) = 1.46, p = 0.61 in POP-SOS-WT. (TIF) (TIF) [file pone.0120753.s001.tif]

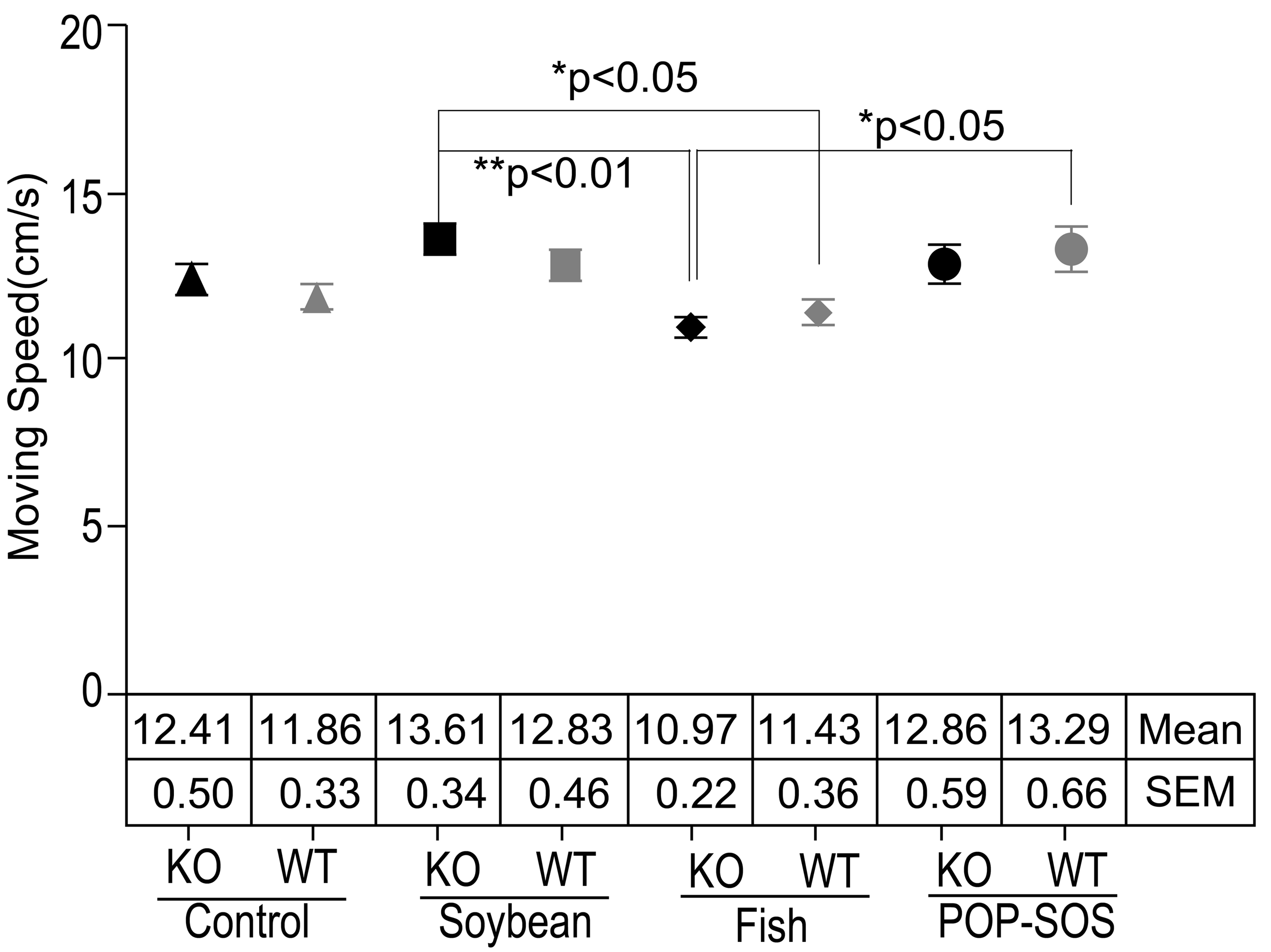

Supplement: S2 Fig — Moving speed (cm/s) at the first entry was extracted from Fig. 1. Statistical analysis was performed by ordinary one-way ANOVA with Tukey’s multiple comparison test for comparison of the 2 genotypes that ate foods containing each type of oils: F(7, 55) = 3.59, **p = 0.003; Tukey’s: **p = 0.007 [fish-KO with soybean-KO], *p = 0.049 [fish-WT with soybean-KO], and *p = 0.035 [fish-KO with POP-SOS-WT]. (TIF) (TIF) [file pone.0120753.s002.tif]

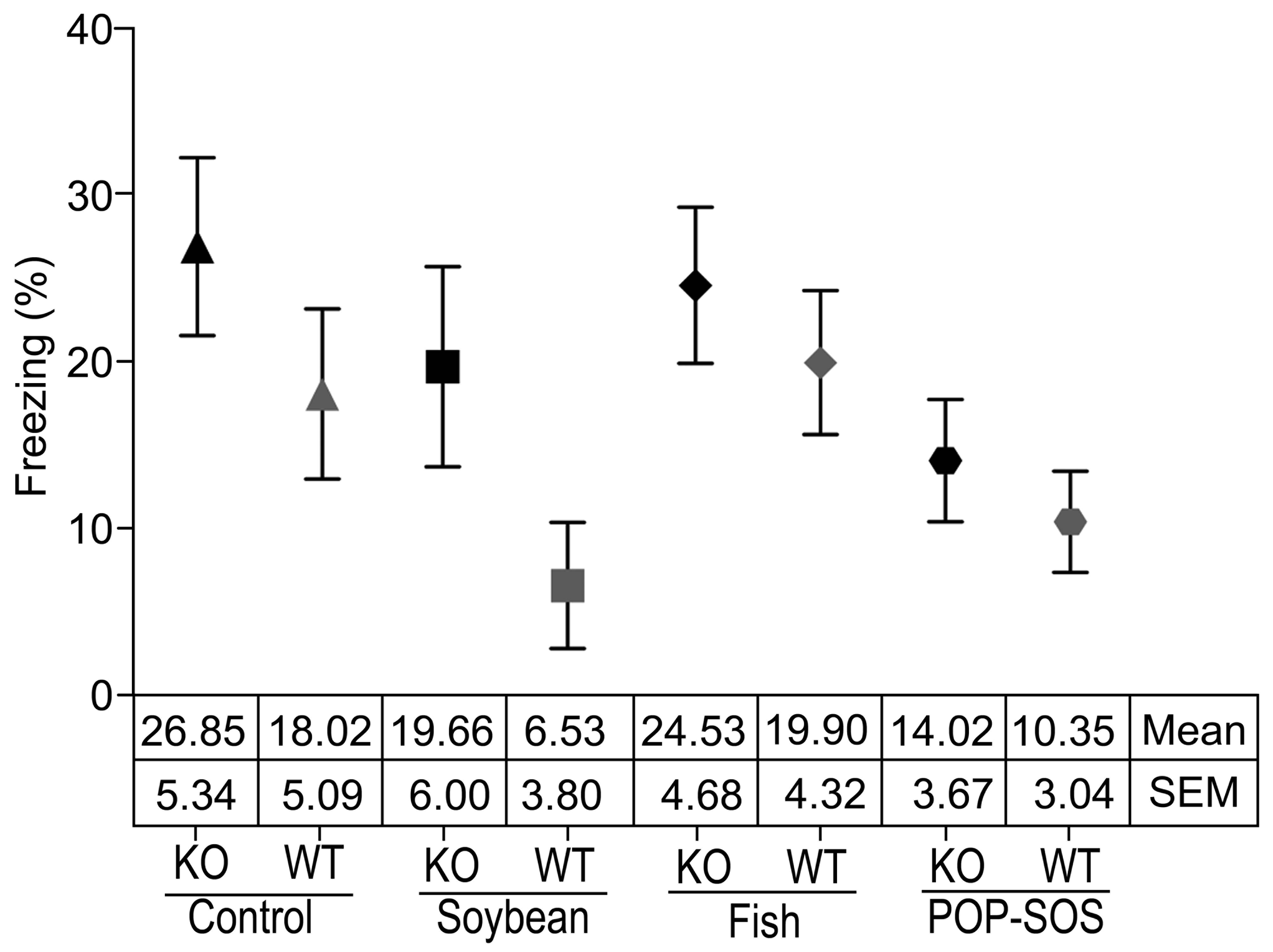

Supplement: S3 Fig — Mice received training stimuli coupled with tone and electrical shock twice in a chamber where the mouse was resting on day 1. Trials were 3 min in length and the last 1.5 min of each trial was analyzed. Statistical analysis was performed by ordinary one-way ANOVA with Tukey’s multiple comparison tests to compare the 2 genotypes that ate foods containing each type of oils: F(7, 142) = 2.11; *p = 0.047, no significance in Tukey’s. (TIF) (TIF) [file pone.0120753.s003.tif]

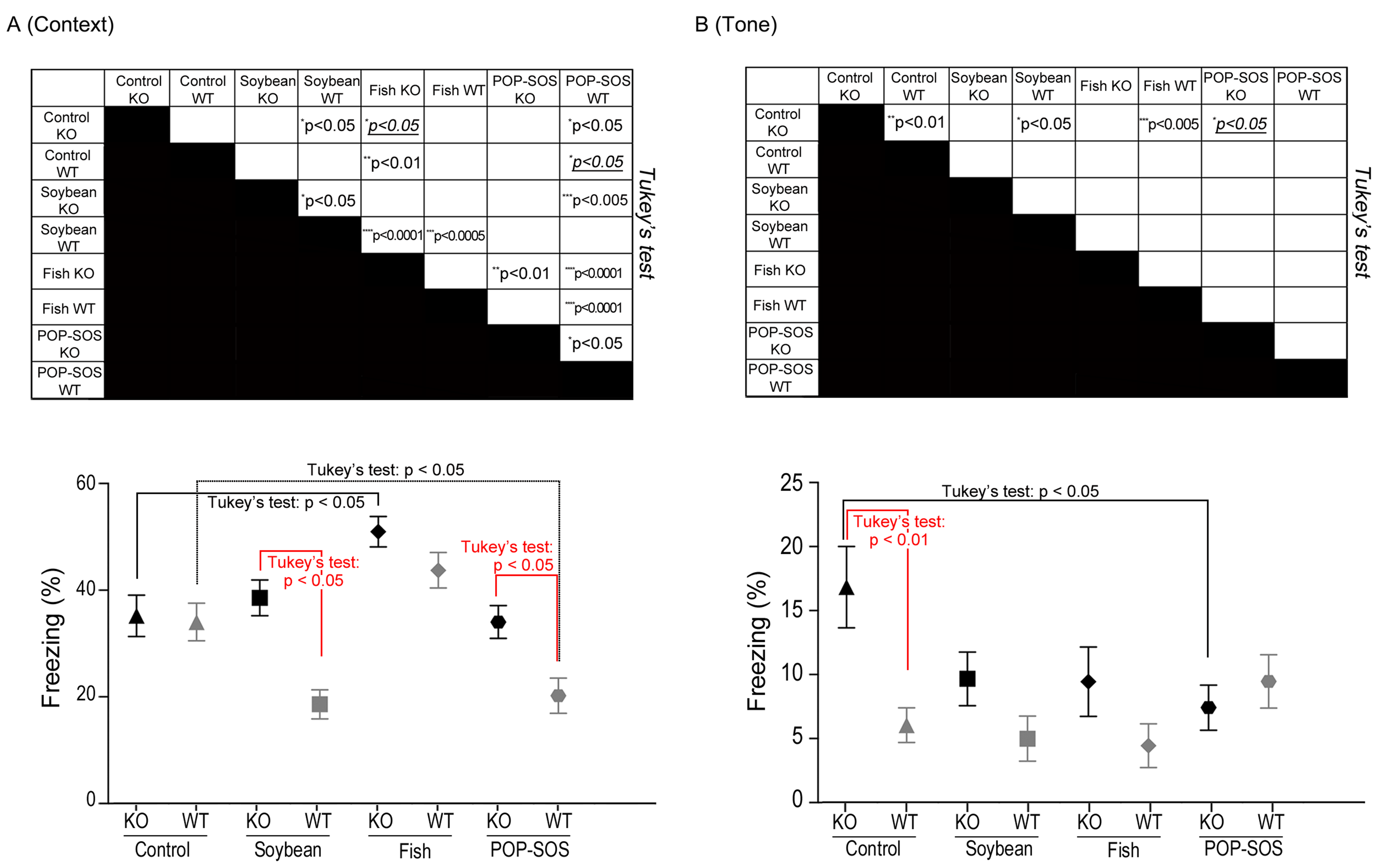

Supplement: S4 Fig — Graphs of context freezing (%) (A) and tone freezing (%) (B) shown in Fig. 2 were gathered. Statistical analysis was performed by ordinary one-way ANOVA to compare the 2 genotypes on consumption of food containing each type of oil: F(7, 392) = 9.84, ****p < 0.0001 in A; F(7, 242) = 3.12, **p = 0.004 in B. Significant difference by Tukey’s multiple comparison test is shown in the tables presented above the figures. The p value for Tukey’s test in mice that ate any oil-rich diet compared to mice that ate a control diet is underlined and written in bold font. Differences identified with Tukey’s test between any oil-rich diet and the control diet are shown with a large-shaped line (ST3Gal IV-KO mice) and a large dotted line (WT mice). Significant difference by Tukey’s test between ST3Gal IV-KO and WT mice that ate the same diet is denoted with a red line. (TIF) (TIF) [file pone.0120753.s004.tif]
