# Supplementary material for: Differential Effects of Dietary Oils on Emotional and Cognitive Behaviors
Source: PLoS One. 2015 Mar 23;10(3):e0120753. doi: 10.1371/journal.pone.0120753 (PMC4370753; doi:10.1371/journal.pone.0120753)
Supplement: S1 File — (DOCX) [file pone.0120753.s005.docx]

**Supporting Information**

**S1 File. Effects of oil-rich diet on contextual and tone fear tests.**

Statistical values that were analyzed in Fig. 3. While significant values obtained by two-tailed unpaired t-test are described in Fig. 3 legend, values showing no difference by unpaired t-test are exhibited below. Fig. 3A shows the two-tailed unpaired t-test in which $ shows statistical significance between WT and KO mice (red): F(27, 23) = 1.67, p = 0.39 in control; F(15, 19) = 1.52, ^$^p = 0.047 in soybean; F(23, 23) = 1.25, p = 0.79 in fish; F(31, 31) = 1.08, p = 0.44 in POP-SOS. Fig. 3B shows the two-tailed unpaired t-test (red): F(27, 23) = 2.49, p = 0.27 in control; F(15, 19) = 1.43, ^$$^p = 0.010 in soybean; F(23, 23) = 2.10, ^$^p = 0.011 in fish; F(31, 31) = 1.26, p = 0.10 in POP-SOS. #Hashes denote statistical significance obtained using the two-tailed unpaired t-test between the first and second days. F(23, 23) = 1.26, ^#^p < 0.0001 in control-KO; F(27, 27) = 1.87, ^#^p = 0.003 in control-WT; F(19, 19) = 1.08, p = 0.35 in soybean-KO; F(15, 15) = 1.01, p = 0.95 in soybean-WT; F(23, 23) = 2.86, ^#^p = 0.002 in fish-KO; F(23, 23) = 1.09, p = 0.26 in fish-WT; F(31, 31) = 1.120, ^#^p = 0.024 in POP-SOS-KO; F(31, 31) = 1.52, p = 0.24 in POP-SOS-WT.
