# Supplementary material for: Differential Effects of Dietary Oils on Emotional and Cognitive Behaviors
Source: PLoS One. 2015 Mar 23;10(3):e0120753. doi: 10.1371/journal.pone.0120753 (PMC4370753; doi:10.1371/journal.pone.0120753)
Supplement: S1 Table — (DOCX) [file pone.0120753.s006.docx]

| Diet | Control diet | | Soybean oil diet | | Fish oil diet | | POP-SOS diet | |
| --- | --- | --- | --- | --- | --- | --- | --- | --- |
| Percentage of oil | AIN93G containing  7% soybean oil^$^ | | AIN93G containing  20% soybean oil | | AIN93G containing  2% soybean oil^&^ and  18% fish oil | | AIN93G containing  2% soybean oil^&^ and  18% POP-SOS triglycerides | |
| Ingredients | weight (g) | kcal/100 g^#^ | weight (g) | kcal/100 g | weight (g) | kcal/100 g | weight (g) | kcal/100 g |
| Casein | 100.2 | 67.62 | 100.2 | 67.62 | 100.2 | 67.62 | 100.2 | 67.62 |
| l-Cysteine | 1.5 | 1.20 | 1.5 | 1.20 | 1.5 | 1.20 | 1.5 | 1.20 |
| β cornstarch | 198.5 | 139.70 | 33.5 | 23.58 | 33.5 | 23.58 | 33.5 | 23.58 |
| α cornstarch | 66.0 | 46.45 | 66.0 | 46.45 | 66.0 | 46.45 | 66.0 | 46.45 |
| AIN93G vitamin mix | 5.0 | 4.00 | 5.0 | 4.00 | 5.0 | 4.00 | 5.0 | 4.00 |
| AIN93G mineral mix | 17.5 | 3.08 | 17.5 | 3.08 | 17.5 | 3.08 | 17.5 | 3.08 |
| Choline bitartrate | 1.25 | 0 | 1.25 | 0 | 1.25 | 0 | 1.25 | 0 |
| Tertiary butylhydroquinone | 0.0096 | 0 | 0.0096 | 0 | 0.0096 | 0 | 0.0096 | 0 |
| Soybean oil^##^ | 35.0 | 63.0 | 100.0 | 180.0 | 10.0 | 18.0 | 10.0 | 18.0 |
| Fish oil^*^ | - | - | - | - | 90.0 | 162.0 | - | - |
| POP-SOS^**^ | - | - | - | - | - | - | 90.0 | 162.0 |
| Sucrose | 50.0 | 39.68 | 50.0 | 39.68 | 50.0 | 39.68 | 50.0 | 39.68 |
| Cellulose powder | 25.0 | 0 | 125.0 | 0 | 125.0 | 0 | 125.0 | 0 |
| Total | 500.0 | 364.74 | 500.0 | 365.61 | 500.0 | 365.61 | 500.0 | 365.61 |

^#^Calories were calculated with 4 kcal for protein and carbohydrate, and 9 kcal for oil.

^$^AIN93G contained 7% soybean oil, which included 1.6% oleic acid [(number of carbons in the fatty acid) : (number of double bonds in the fatty acid chain), 18:1, n-9], 3.7% linoleic acid (18:2, n-6), and 0.5% linolenic acid (18:3, n-3) (standard AIN93G).

^##^Soybean oil contained 4.7% oleic acid, 10.6% linoleic acid, and 1.5% linolenic acid.

^&^Feeds except for control contained 2% soybean oil to protect against essential oil deficiency.

^*^Fish oil: sardine oil containing 28% EPA and 12% DHA and bonito oil containing 24% DHA (22:6, n-3) and 5% EPA (20:5, n-3) were used as ingredients of diet. Finally, fish diet contained 3.2% DHA and 3.1% EPA.

^**^POP-SOS contained triglycerides that were mixed at a ratio of POP:SOS = 1:1 and finally contained 6.7% of palmitic acid (16:0), oleic acid, and stearic acid (18:0) each.
