# Supplementary material for: Differential Effects of Dietary Oils on Emotional and Cognitive Behaviors
Source: PLoS One. 2015 Mar 23;10(3):e0120753. doi: 10.1371/journal.pone.0120753 (PMC4370753; doi:10.1371/journal.pone.0120753)
Supplement: S2 Table — (DOCX) [file pone.0120753.s007.docx]

| C:D | Fatty acids | | | C57Bl/6J mouse | | C57Bl/6 mouse | | AIN93G | | AIN93G | | AIN93G | | AIN93G | |
| --- | --- | --- | --- | --- | --- | --- | --- | --- | --- | --- | --- | --- | --- | --- | --- |
| (omega)^#^ |  | | | brain fatty acids | | brain phospholipid | | containing | | containing | | containing | | containing | |
|  |  | | | (%)^1)^ | | fatty acids | | 7% soybean oil | | 20% soybean oil | | 2% soybean oil | | 2% soybean oil | |
|  |  | | |  | | (%)^2)^ | | (%)^3)^ ^$^ | | (%)^$^ | | 18% SOS-POP (%)^$^ | | 18% fish oil (%)^$^ | |
| 14:0 | Myristic acid | | | n.d. | | n.d. | | 0.01 | | 0.02 | | 0.00 | | 0.83 | |
| 16:0 | Palmitic acid | | | 27.00± 2.09 | | 25.91 ± 0.80 | | 0.74 | | 2.12 | | 6.91 | | 2.65 | |
| 16:1 (n-7) | Palmitoleic acid | | | n.d. | | n.d. | | 0.01 | | 0.02 | | 0.00 | | 1.37 | |
| 18:0 | Stearic acid | | | 21.32 ± 4.22 | | 19.51 ± 0.21 | | 0.30 | | 0.86 | | 6.79 | | 0.59 | |
| 18:1 (n-9) | Oleic acid | | | 20.36 ± 5.19 | | 18.47 ± 0.48 | | 1.65 | | 4.70 | | 7.17 | | 2.17 | |
| 18:2 (n-6) | Linoleic acid | | | 4.64 ± 3.46 | | n.d. | | 3.75 | | 10.70 | | 1.07 | | 1.27 | |
| 18:3 (n-3) | Alpha-linolenic acid | | | n.d. | | n.d. | | 0.46 | | 1.32 | | 0.13 | | 0.21 | |
| 18:4 (n-3) | Stearidonic acid | | | n.d. | | n.d. | | 0.00 | | 0.00 | | 0.00 | | 0.41 | |
| 20:0 | Arachidic acid | | | n.d. | | n.d. | | 0.03 | | 0.08 | | 0.01 | | 0.05 | |
| 20:1 (n-9) | Gadoleic Acid | | | n.d. | | 2.01± 0.15 | | 0.01 | | 0.04 | | 0.00 | | 0.10 | |
| 20:4 (n-6) | Arachidonic acid (AA) | | | 7.72 ± 2.65 | | 9.32 ± 0.35 | | 0.00 | | 0.00 | | 0.00 | | 0.27 | |
| 20:5 (n-3) | Eicosapentaenoic acid (EPA) | | | n.d. | | n.d. | | 0.00 | | 0.00 | | 0.00 | | 3.21^&^ | |
| 22:5 | Docosapentaenoic acid | | | n.d. | | 0.11 ± 0.02 (n-6) | | 0.00 | | 0.00 | | 0.00 | | 0.36 (n-3) | |
| 22:6 (n-3) | Docosahexaenoic acid (DHA) | | | 12.47 ± 4.61 | | 15.30 ± 0.95 | | 0.00 | | 0.00 | | 0.00 | | 3.51^&^ | |

n.d. not determined. #C is the number of carbon atoms in the fatty acid and D is the number of double bonds in the fatty acid.

Compositions of diets including dietary oil are shown in Table S1. $% means each fatty acid per 100g diet.

^&^Batch of fish oil in Table S2 is different from batch that mice that received behavioral tests fed in Table 1.

1) Reference 1 indicates the brain fatty acid composition in female C57Bl/6j mice, 9 weeks of age (n = 5 or 6).

2) Reference 2 indicates brain phospholipid fatty acid composition in male C57Bl mice, 7 weeks of age (n = 8).

3) Reference 3 indicates compositions of free fatty acids in soybean oil, which was used for compositions of soybean oil.

References:

1. Yu H, Bi Y, Ma W, He L, Yuan L, et al. (2010) Long-term effects of high lipid and high energy diet on serum lipid, brain fatty acid composition, and memory and learning ability in mice. Int J Dev Neurosci 28:271-276.
2. Levant B, Ozias MK, Guilford BL, Wright DE (2013) Streptozotocin-induced diabetes partially attenuates the effects of a high-fat diet on liver and brain fatty acid composition in mice. Lipids. 48: 939-948.
3. Standard Tables of Food Composition in Japan, fifth revised and enlarged edition: (2005) - Fatty Acids Section - Fats and Oils in Table 1. Fatty Acid composition.  Subdivision on Resources, The Council for Science and Technology, Ministry of Education, Culture, Sports, Science, and Technology, Japan.
